# Risk Factors Associated with Intimate Partner Violence against Chinese Women: A Systematic Review

**DOI:** 10.3390/ijerph192316258

**Published:** 2022-12-05

**Authors:** Qian Zhao, Yuxin Huang, Mei Sun, Ying Li, Lisa L. Lommel

**Affiliations:** 1 School of Nursing, Anhui Medical University, 15 Feicui Road, Hefei 230601, China; 2 XiangYa Nursing School, Central South University, 172 Tongzipo Road, Changsha 410013, China; 3 Teaching and Research Section of Clinical Nursing, XiangYa Hospital of Central South University, 87 Xiangya Road, Changsha 410008, China; 4 XiangYa Center for Evidence-Based Practice & Healthcare Innovation: A Joanna Briggs Institute Affiliated Group, Changsha 410013, China; 5 School of Nursing, University of California San Francisco, 2 Koret Way, San Francisco, CA 94143-0606, USA

**Keywords:** China, intimate partner violence, systematic review, risk factors, women

## Abstract

Background: The prevalence of intimate partner violence against women in China remains high. Understanding associated risk factors will help inform prevention. The purpose of this systematic review was to identify associated risk factors of intimate partner violence against women in mainland China. Methods: Nine English and Chinese databases were searched from 1 August 2008–2 August 2022. Reference lists of relevant studies supplemented the initial results. The Joanna Briggs Institute (JBI) Critical Appraisal Checklist for Studies Reporting Prevalence was used to assess article quality. Study results were combined in a narrative synthesis. Results: Nineteen eligible studies were identified. Examples of key intimate partner violence risk factors included: partner’s low education or income, unhealthy habits (gambling), women’s marital status, poor health or education, women’s or partner’s childhood abuse or witnessing thereof at home, or multiple children and husband dominance. Conclusions: Despite the significant changes in Chinese policies and the new law, IPV continues, and this review has highlighted vulnerable women who need identification and protection. Further study is needed of individual (e.g., psychological well-being), relationship/family, and society/cultural variables.

## 1. Introduction

Intimate partner violence (IPV) is defined as an act of physical assault, psychological abuse, sexual abuse, and/or other controlling behaviors perpetrated by an intimate partner [1]. In China, domestic violence refers to physical abuse, emotional abuse, sexual abuse, or any controlling behaviors by family members or between spouses [2].

IPV has become an important public health and social issue. Globally, the prevalence and severity of IPV is higher against women than men [3]. It is estimated that one in three women have experienced violence within their intimate partner relationships [4]. A scoping review reported that the life-time prevalence of physical, psychological, and sexual violence against women in mainland China was estimated to be 2.5–5.5%, 17.4–24.5%, and 0.3–1.7%, respectively [5]. Studies demonstrate that IPV against women endangers their self-esteem and autonomy [6,7]. IPV is also associated with a variety of adverse mental and physical health outcomes, such as an enhanced risk for injury, sexually transmitted infections/HIV, post-traumatic stress order, depression, anxiety, and suicidal thoughts [8,9].

With an increasing awareness of adverse effects of IPV, more attention is being paid to women who have experienced it. In 2013, the United Nations Commission on the Status of Women released the agreed conclusion of a global call to take action to eliminate all forms of violence against women and emphasized the significance of prevention of violence against women [10]. In China, which is influenced by a traditional patriarchal culture, IPV is often regarded as a private family matter [11]. As a result, IPV is often over-looked [12]. However, with mainland China’s rapid economic development over the past 20 years, traditional family structures and gender role concepts have been altered, which has influenced power interactions in intimate relationships [13]. In July 2008, seven ministries and commissions, including the All-China Women’s Federation, issued several opinions focused on preventing and curbing domestic violence, thus marking an important milestone in China’s efforts to influence domestic violence [14]. In March 2016, China’s first law on IPV, the Anti-Domestic Violence Law of the People’s Republic of China, was officially implemented in mainland China [2]. With these political and legal changes, the status and protection of women in China increased [13].

Risk factors that have a positive effect, although associated with a greater likelihood of IPV victimization and perpetration, are not the cause of IPV. According to the ecological model of IPV, a combination of individual, relational, community, and social factors contribute to the risk of being a victim or perpetrator of IPV [15,16]. Understanding these multilevel factors can help target prevention interventions. Furthermore, in order to decrease the prevalence of IPV, it is important to understand the risk factors associated with these harmful behaviors. To the best of our knowledge, the last systematic review focusing on IPV risk factors was published in 2008 [13]. This review demonstrated that demographic factors including low socio-economic status, poor education of either partner, or growing up in a rural area were associated with increased risk of IPV against women [13]. Personal behaviors including alcoholism, smoking, and illegitimate drug use were also associated with an increased risk of IPV against women [13]. Relationship factors including long duration of marriage, marital conflict, unsatisfactory marital quality, power/status disparity, extramarital affairs, and sexual jealousy were linked to an increased risk of IPV against women [13]. Finally, social factors including insufficient social support, patriarchal beliefs and IPV justification were all associated with IPV against women [13]. Although there is clarity regarding risk factors for IPV against Chinese women prior to 2008, the significant changes in Chinese policies in 2008 and 2016 make it essential to re-examine associated factors [2,14]. Therefore, this systematic review focuses on published research from 2008 to determine risk factors associated with IPV against women in mainland China in recent times.

## 2. Methods

The Preferred Reporting Items for Systematic Reviews (PRISMA) Checklist [17] and the Joanna Briggs Institute (JBI) guidelines [18] were used to conduct this systematic review. Covidence software was used to manage and streamline the data.

### 2.1. Data Sources and Search Strategy

A literature search of English- and Chinese-language publications was conducted from 1 August 2008 through 2 August 2022 with two science librarians’ support (one in mainland China and one in the US). Five English electronic databases (PubMed, Web of Science, PsychINFO, Sociological Abstracts and Embase), four Chinese electronic databases (China National Knowledge Infrastructure [CNKI], VIP, Wanfang Data, and China Biology Medicine disc [CBMdisc]), and “related links” in PubMed were searched. Search terms in Chinese and English were used for each language search as appropriate. The following search strategy were used (see Table 1). Selected articles’ reference lists were manually screened to identify additional publications.

### 2.2. Inclusion/Exclusion Criteria

Studies were included if they: (1) were in English or Chinese, (2) were related to mainland Chinese women, (3) reported on women experiencing IPV, (4) were cross-sectional, case-control, or cohort studies’ results; or (5) were about a prevalence estimation or risk factors of IPV victimization.

Studies were excluded if they were: (1) focused on specific population groups including pregnant women, sex workers, women with mental illness, women seeking abortion, women with special circumstances (e.g., disabled, HIV, post-earthquake), same-sex couples or perpetrators, or (2) examined children, elders, or university/college dating violence. We excluded opinions, editorials, guidelines, and unpublished manuscripts (e.g., thesis and dissertations).

### 2.3. Quality Assessment

Article quality was evaluated using the JBI Critical Appraisal Checklist for Studies Reporting Prevalence Data [18]. Information such as sample size and appropriateness of the managed analysis was considered in the nine-item tool. Each item was appraised as yes, no, unclear or not applicable. Based on overall appraisal and author consensus, each study was placed in one of three categories: include, exclude, seek further information. Each article was reviewed by two researchers independently using the tool to lessen bias, and discrepancies were settled by discussion or by seeking advice from a third nurse-scientist. There were no disputes on the included studies.

### 2.4. Data Extraction

The JBI Data Extraction Form for Prevalence and Incidents Studies [18] was used by two reviewers to extract data from the included studies. The parameters were extracted as follows: author(s), publication year, journal, research design, location, setting, sample size, measurements, duration of data collection, age, education, definition of IPV, victims, perpetrators, and variables associated with IPV victimization. Consensus was reached through team discussions.

## 3. Results

### 3.1. Search Results

**Figure 1:**
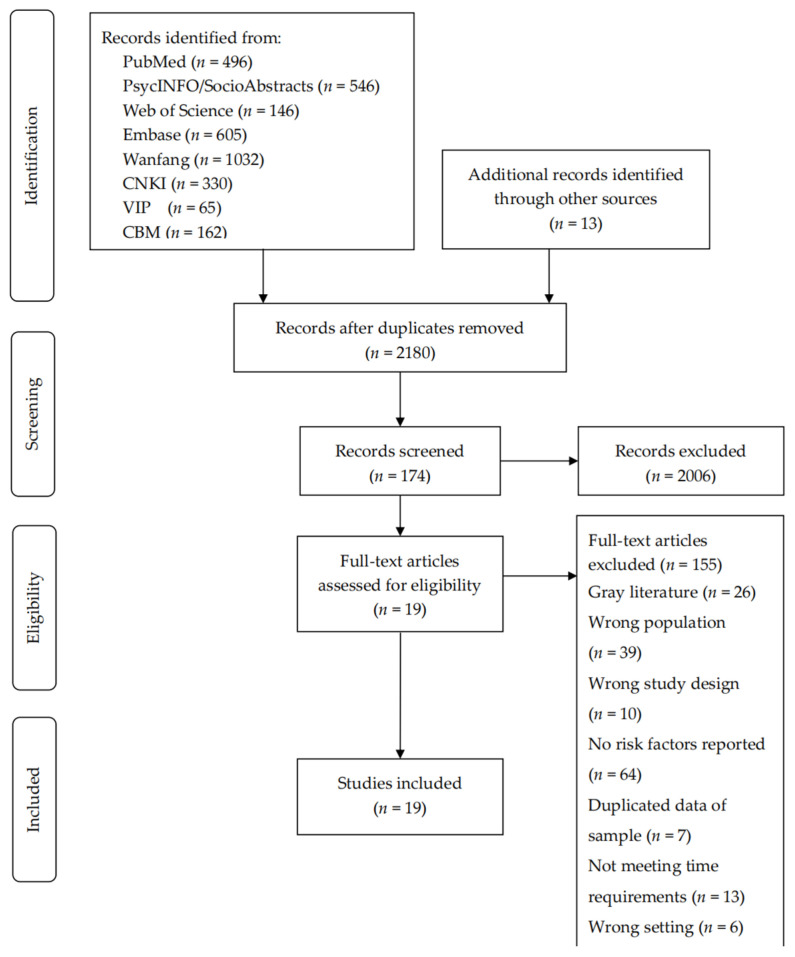
Prisma flow diagram of search results.

The search yielded 3395 papers. Following the removal of duplicates, 2180 papers remained. After examining titles and abstracts for self-reported IPV prevalence or risk factors of IPV victimization, 174 abstracts remained. We further examined these abstracts for eligibility according to inclusion/exclusion criteria. Finally, 17 selected full-text articles were read and reference lists were hand-searched. Two articles were added for a total of 19 studies that met search criteria and the aim of this systematic review (see Figure 1).

### 3.2. Characteristics of Studies Included

All 19 studies used a cross-sectional design and were published from 2010–2021 (see Table 2). Six of the 19 studies were conducted nationally in mainland China, whereas 13 studies were regional. Study participants ranged in age from 16–65 years. In 16 studies, female victims of IPV were married or divorced. Five of the 16 studies were secondary data analyses of The Third Wave of China’s Women Social Status Survey [19]. Two other studies used the database of a survey conducted in a large city in southern China. Sample sizes ranged from 194 to 36,023 participants. In five studies, the authors used a combination of both administrative databases and questionnaires, whereas 14 used only a questionnaire. Eleven of the 14 studies reported questionnaire response rates and all were >70% (except one, which varied from 43% to 60% depending on location). In 19 studies, fourteen studies used the revised Conflict Tactics Scales (CTS2) [20], a modified version of CTS2 or the short form of the revised Conflict Tactics Scales (CTS2s) [21] as measurement scales. Four of the 19 studies used a self-administered questionnaire. The remaining one study used a Questionnaire based on WHO Multi-country Study on Women’s Health and Domestic Violence against Women Health and Life Experiences Questionnaire [22]. Of the 19 studies, 16 studies evaluated whether women experienced IPV as experiencing any physical, psychological, or sexual violence; two studies defined women’s IPV as experiencing either physical or psychological violence; and one study defined women’s IPV as experiencing non-joking physical violence (see Table 2).

### 3.3. Associated Risk Factors

Most studies used logistic regression models with a 0.05/0.1 significance level. Two studies used probit regression models with a 0.01 significance level and a third used confirmatory factor analysis with a 0.05 significance level. Risk factors were assessed at an individual (female victims/male perpetrators) level, relationship/family level, and society/cultural/attitude level (see Table 3).

#### 3.3.1. Individual-Associated Risk Factors (Female Victims)

In 16 studies that considered age, two found that older age was positively associated with IPV victimization for Chinese women, whereas two others noted that younger age was positively correlated with IPV victimization. Fourteen studies examined the participants’ education level; nine found women with a low education had a significantly higher associated risk factor for IPV victimization, whereas two found that women with a high education were more correlated with IPV victimization. In addition, employed women were found to be more likely to suffer from IPV in 3/5 studies that considered this factor. A woman’s marital status (cohabitation, remarriage, divorce or in the divorce process) was positively associated with IPV victimization in 5/6 studies that included this factor. (Table 3).

Four studies included local hukou (household registration) status and three of these indicated that belonging to the floating (migrating) population was positively correlated with IPV victimization for women. Two studies considered land-rights status and suggested that women with no claim to contract or residential land were more likely to suffer from IPV. Health status had a negative significant association with IPV victimization for women in all four articles that included this factor. Strong feelings of loneliness or helplessness had a significant positive association with IPV victimization for women in the two studies that included this factor (Table 3).

#### 3.3.2. Individual-Associated Risk Factors (Male Perpetrators)

The education level of women’s intimate partners was identified in seven articles and showed a negative significant association with IPV victimization of women in three studies. Both articles that considered the income level of women’s intimate partners, showed it was negatively associated with IPV victimization. Factors related to husbands’ unhealthy habits, i.e., gambling, alcohol use, or drug use, were included in five studies and four studies showed a positive significant correlation with IPV victimization. If a wife’s economic contribution to a family was higher compared to her husband’s contribution, this was found to be positively associated with IPV victimization (increased risk) in the two studies that considered this factor. Nonetheless, 2/3 studies that reported the husband’s economic contribution to a family as higher than his wife’s, found this was positively associated with IPV victimization as well (increased risk) (Table 3).

#### 3.3.3. Relationship/Family-Associated Risk Factors

Three studies reported that women who had experienced family-of-origin violence or whose intimate partners had experienced family-of-origin violence were more likely to experience IPV victimization. Women who have been married longer were found to be more likely to suffer from IPV in 3/8 studies that considered this factor. The number of children in a family was considered to have a positive significant correlation with IPV victimization of the women in 2/5 studies that took this factor into consideration. In the two studies that considered family size, one reported it was negatively associated with IPV victimization, whereas the other reported it was positively associated with IPV victimization. Factors related to social support were considered in three studies and had a negative significant correlation with IPV victimization in two of these studies. (Table 3).

#### 3.3.4. Society/Cultural/Attitude-Associated Risk Factors

Song and Zhang considered the disparity in sex composition in the marriage market and found that a comparative excess of men in a community was correlated with a greater probability of IPV victimization of women [27]. The level of understanding of the Women’s Rights Protection Law was included in one study and suggested that women with a lower level were more likely to be IPV victims [26]. More importantly, patriarchy-related factors (including husband dominance, identification with traditional family culture/gender role and IPV justification) were taken into account in six studies and all had a positive association with IPV victimization (Table 3).

## 4. Discussion

This systematic review identified risk factors most often associated with IPV against women in mainland China. These included individual factors (e.g., low education or income level) and relationship/family (e.g., marital status, family history), as well as social, cultural and attitudinal factors (e.g., loneliness, patriarchy ideology). Other risk factors, such as a couple living with the husband’s parents, a high level of marital conflict, disparity in sex composition in the marriage market and a woman’s understanding of the Women’s Rights Protection Law or Domestic Violence Law were only included in the final selected articles once or were not found to be significant and therefore yielded scant evidence.

Our results draw attention to the impact of a low education level on a woman’s risk for IPV in China. Most studies in China showed that women who have suffered IPV have a little education, which is also reported by other studies worldwide [41]. Education disparity between partners was considered in one included study [26], which reported that a woman with a higher education than her intimate partner was correlated with psychological violence. A study in Ghana found that a husband whose education level was lower than his partner’s and who lacked a dominant position might achieve control through psychological violence to gain the dominant position in the family [42].

Our results also demonstrate that a male partner with a low-education level or a low-income level is associated with women’s IPV victimization, which is consistent with a previous systematic review of IPV in asylum seekers and refugees [43]. In addition, a study examining 37-years of successive IPV data in China suggested that men with lower levels of education were more likely to perpetrate violence against their female partners [44]. According to feminist theory, male partners’ low education and income may show they are short of male power; this may seduce the men to exert violence over their female partner to recover their power within the relationship [45]. Overall, there is still a lack of research on male perpetrators in violence against women studies. More research is still needed to understand the risk factors for male perpetrators of violence, such as alcohol abuse, smoking, etc.

Employment in this study was a risk factor for women to experience IPV, which is inconsistent with previous studies in Western nations [46]. A study using typical data from 31 low- or middle-income countries to investigate the correlation of IPV with unemployment found that increased violence was correlated with an improvement in women’s employment opportunities in nations in which women have more restricted access to divorce than men [47]. Since patriarchal norms are much more powerful in most low- or middle-income countries, inequality in access to divorce remains. Women’s employment is increasing and producing a potential mismatch between economic variations and norms, which may also increase the likelihood of violence [48].

The results of our study also highlight that in mainland China, marital status (cohabitation, remarriage, divorced or in the divorce process) is related to IPV against women. There is evidence that cohabiting women are particularly vulnerable for IPV [49], potentially because they may have a low income and education, unstable employment status and are younger when initiating a relationship [50].

The results of our study indicated that a Chinese woman’s low-health status was associated with IPV victimization. A study from Korea [51] found that women who have experienced IPV often rated their general health as less than excellent and more likely as fair or poor, which is congruous with our results. Practitioners should be aware that IPV victimization may be related to a woman’s health status and access to medical care [52].

Our results show that Chinese women who have no claim to contract or residential land face a significantly higher risk of being psychologically or physically abused by their male intimate partners than women who have such claims. Other studies have reported that holding assets (such as land, housing) alone or in combination with a male partner may contribute to a reduced incidence of IPV against women depending on the context [53]. More supportive contexts of women’s rights (e.g., social norms and institutions) were more likely to contribute to positive outcomes and reduced IPV [54]. However, there is limited research on this topic.

We found that mainland Chinese women who belonged to the floating population were more likely to report IPV victimization. The majority of migrants in China face negative situations of poverty, high work stress, low social status, etc. [55]. Moreover, long periods of family or partner separation have to be confronted by many migrants. Many studies have suggested that these circumstances are recognized associated risk factors for IPV [10].

In consensus with previous studies conducted in North America and other high-income countries [56], our results reveal that gambling problems are also an important associated risk factor for male IPV in mainland China. A systematic review by Dowling et al. demonstrated a significant relationship between gambling and both IPV victimization and IPV perpetration [56]. However, further research is needed to investigate the involvement of possible mediating and moderating variables in the relationship between IPV and problem gambling.

In addition, our results found that the relative household economic contribution of women was correlated with IPV victimization. According to the gendered resource theory [57], the influence of relative resources (e.g., educational attainment, income) is based on a husbands’ gender ideologies. Studies [47] have indicated that if husbands adhere to traditional gender-role expectations (husband as chief breadwinner), their wives’ portion of relative household economic contributions is positively associated with IPV victimization (increased risk).

The risk of women’s IPV victimization is increased owing to witnessing or experiencing family-of-origin violence [58,59]. Feminist theorists argue that IPV is more likely to be present among women whose intimate partners have adapted to their families of origin, neighborhoods, and society to encouragingly approve of violence using in intimate relationships, and adopt traditional male-dominant expectations [57]. Witnessing or experiencing family-of-origin violence can be regarded as a research area to support the interpretation of the feminist-informed theory of IPV.

Our results also show that having multiple children is correlated with IPV. Based on the prior literature, mothers with more children have fewer choices to leave or end intimate relationships with a partner with a history of IPV than mothers with no children and fewer children [60]. This is due to the better psychological and behavioral development of the child and the woman’s fears of social and financial pressure about raising the child alone in the future. Unfortunately, there is no systematic review that has pointed out clear evidence of the relationship between number of children and IPV in mainland China.

Feelings of loneliness and helplessness were positively correlated with IPV victimization of mainland Chinese women in our systematic review, which is consistent with another study from the US [61]. According to Baumeister, Smart and Boden’s postulation, violence can be induced by high self-evaluation amalgamated with a self-esteem threat [62]. Chinese women with high levels of self-esteem may be less willing to adjust their self-evaluation, and are therefore more likely to constitute a threat to male authority and privilege established in Chinese society [33]. Further studies are needed to evaluate the relationship between women’s likelihood of IPV experiences and their psychological well-being by considering possible mediating effects.

Male dominance, inequitable gender roles, and IPV justification were revealed as significant factors for IPV, which is consistent with previous studies. An earlier study showed that women who endorsed inequitable gender roles and male dominance were more willing to endure violence and considered it as a private issue [11]. Other studies (including studies conducted in China) argued that women who approved of female dominance or egalitarianism increased the likelihood that they would experience IPV victimization, especially physical and/or psychological violence [28,63]. Further research is needed to explain the connection of female dominance or egalitarianism and IPV victimization in a Chinese context.

Compared to the review of IPV against women published in 2008 [13], this systematic review identified more personal risk factors associated with IPV, including poor health, growing up in a Western area, an imbalance in household economic contributions between partners, lack of land rights or floating status, more online activity, and greater feelings of loneliness or helplessness. Additional family and social risk factors were also identified, including women’s or partner’s childhood abuse or witnessing thereof at home, multiple children and poor knowledge of Women’s Rights Protection Law. Additionally, most of the risk factors are consistent with previous studies and provide a valuable reference for the prevention of male-on-female intimate partner violence in mainland China.

## 5. Conclusions

In the 19 selected articles for this study, little consideration was given to other important IPV risk factors, such as couples living with the husband’s parents, the disparity in sex composition in the marriage market, and the level of woman’s understanding of the Chinese Women’s Rights Protection Law or Domestic Violence Law.

The main risk factors that correlated with IPV victimization of Chinese women were identified in this systematic review. Despite the significant changes in Chinese policies and the new law, IPV continues, and this review has highlighted vulnerable women who need identification and protection. These include women with partners who have low education or income, better original family economic status, or unhealthy habits (gambling), or women who are employed, have low health or education, have no land, are members of the floating population, make a larger economic contribution than their partners, and experience loneliness/helplessness. Other factors include a women or partner’s history with childhood abuse or witnessing thereof at home, multiple children, or husband dominance. Data is missing about psychological well-being risk factors. Further study is needed of individual (e.g., psychological well-being), relationship/family and society/cultural variables.

Several limitations should be considered for this systematic review. First, the self-reporting of IPV victimization infuses the possibility of bias. Second, all studies were cross-sectional, which limits the understanding of causality. Third, this review only included English and Chinese language research. Fourth, mainland Chinese women’s specific groups were excluded, which restricts generalization of our results. Finally, every effort was made to avoid missing relevant data and to conduct a thorough literature review. However, it remains possible that eligible studies were missed because of inadequate indexing or inadequate relevant search terms.

## Figures and Tables

**Table 1:** Search Strategy of studies of intimate partner violence (IPV) against women in mainland China.

Search	Search Terms	Number of PubMed Results
#1	domestic violence OR family violence OR spouse abuse OR spousal abuse OR spouse violence OR spousal violence OR wife abuse OR intimate partner violence OR intimate partner abuse OR intimate violence OR intimate abuse OR partner abuse OR partner violence OR husband violence OR dating violence	17,437
#2	(violence OR abuse) AND (spouses OR spouse OR wife OR husband OR husbands OR boyfriend OR boyfriends)	1678
#3	(battered women OR abused women OR abused woman OR battered woman) AND (husband OR husbands OR spouse OR spousal OR partner OR partners OR boyfriend)	918
#4	Prevalence OR incidence OR epidemiology OR risk factors OR factors	3,470,794
#5	China OR Chinese	2,275,173
#6	#1 OR #2 OR #3	17,903
#7	#4 AND #5 AND #6	496

**Table 2:** Characteristics of English- and Chinese-language studies of intimate partner violence (IPV) against women in mainland China.

Order Source	Location	Setting	Size	Measurement	Survey Year	Definition of IPV	Victim	Perpetrator	Age (Year)	Education (Year)
1 2010 [23]	Hebei	Rural	384	Self-administered questionnaire	Not provided	Experience any type of physical, psychological, and sexual violence	Married women	Former or current husband	Mean ± SD: 38.72 ± 9.29	Junior or lower: 67.4% Senior or higher: 32.6%
2 2011 [24]	Ningxia	Rural	1771	A modified version of CTS2	Current Population Survey 2007	Experience any type of physical assault, injury, sexual coercion, and psychological aggression	Married and divorced women aged 20–64 years	Former or current husband	Mean ± SD: 42.1 ± 10.2	No formal schooling: 51.5%
3 2013 [25]	Mainland China, nationally	Rural and urban	11,040	Questionnaire: Based on reference to CTS2	Nationally Survey 2010	Experience any type of physical, psychological violence, and mandatory controlling behaviors	Married women aged 18–64 years	Former or current husband	Range: 18–64	Mean: 8.8
4 2014 [26]	Mainland China, nationally	Rural and urban	11,093	Questionnaire: Based on reference to CTS2	Nationally Survey 2010	Experience any type of physical, psychological, and sexual violence	Married women aged 18–64 years	Former or current husband	Not provided	Not provided
5 2017 [27]	Mainland China, nationally	Rural and urban	36,023	Questionnaire: Based on reference to CTS2	Nationally Survey 2010	Experience any type of physical and psychological violence	Married women aged 18–64 years	Former or current husband	Mean ± SD: 42.81 ± 10.44	Mean ± SD: 8.54 ± 3.23
6 2015 [28]	Mainland China, nationally	Rural and urban	9605	Questionnaire: Based on reference to CTS2	Nationally Survey 2010	Experience of any type of physical, mental, sexual abuse, and controlling behaviors	Married women aged 18–64 years	Former or current husband	Mean ± SD: 42.54 ± 11.22	Primary and lower: 34% Junior: 38% Senior and higher: 28%
7 2015 [29]	Hunan	Rural	412	CTS2	Current Population Survey 2015	Experience any type of physical, psychological, and sexual violence	Married women aged 20–60 years	Husband	Mean: 38.88	Mean: 9.25
8 2015 [30]	Jiangxi	Rural	483	CTS2	Current Population Survey 2014	Experience of any type of physical, psychological, and sexual violence	Married women migrant workers aged 20–60 years	Husband	20–29: 24.6%; 30–39: 28.2%; 40–49: 29.0%; 50–60: 18.2%	Primary: 37.5% Junior: 39.4% Senior and higher: 23.1%
9 2016 [31]	Beijing	Urban	194	CTS2	Current Population Survey 2006–2007	Experience of any type of physical assault, psychological aggression, and sexual coercion.	Married women aged 20–59 years	Former or current husband	Mean ± SD: 36.6 ± 9.51	Primary and lower: 2.6% Junior: 12.4% Senior and higher: 79.8%
10 2017 [11]	Shanghai	Urban	958	Questionnaire: Based on WHO Multi-country Study [22]	Current Population Survey 2010	Any act of emotional, physical, or sexual abuse by a current or former husband	Married rural migrant women aged 20–49 years	Former or current husband	Mean ± SD: 35.4 ± 6.5	Primary and lower: 34.0% Junior: 50.4% Senior and higher: 15.6%
11 2017 [32]	Mainland China, nationally	Rural	12,374	Questionnaire: Based on reference to CTS2	Nationally Survey 2010	Experience any type of physical, psychological, and sexual violence	Married rural women aged 18–60 years	Former or current husband	Range: 18–60	Not provided
12 2018 [33]	A large city in southern China	Urban	446	Questionnaire: Based on CTS2	Current Population Survey 2013–2014	Experience any of the four types of IPV: physical, psychological, sexual violence, and controlling behaviors	Married and divorced women aged 20–60 years	Former or current husband	Mean ± SD: 38.39 ± 7.84	Mean ± SD: 3.29 ± 0.86
13 2018 [34]	Sichuan	Rural	1501	CTS2s	Current Population Survey 2012	Experience any types of IPV: physical, sexual, and emotional violence	Women aged 16 or older, who had lived locally for at least 2 years	Current or former intimate partner	Mean ± SD: 46.44 ± 13.11	Primary and lower: 74.62% Senior and higher: 7.99%
14 2018 [35]	A large city in southern China	Urban	553	Questionnaire: Based on CTS2	Current Population Survey 2013–2014	Experience any of the four types of IPV: physical assault, psychological aggression, injury, and sexual coercion	Married and divorced women aged 20–60 years	Former or current husband	Mean ± SD: 39.84 ± 8.25	Mean ± SD: 3.17 ± 0.92
15 2019 [36]	Zhejiang	Rural and urban	986	CTS2s	Not provided	Experience of any type of physical, psychological, and sexual violence	Married female migrant workers	Former or current intimate partner	Mean ± SD: 36.59 ± 8.77	Not provided
16 2019 [37]	Mainland China, nationally	Rural and urban	8421	Self-administered questionnaire	National Survey 2000, 2006, 2010, 2015	Experience of being assaulted (not as a joke) by an intimate partner who have/had a sexual relationship with a respondent	Women aged 18–61 years	Former or current intimate partner	2000: 20–64 2006, 2010, 2015: 18–61	Not provided
17 2019 [38]	Wenzhou	Rural and urban	705	CTS2s	Current Population Survey 2018	Experience of any of the three types of IPV: physical assault, psychological aggression, and sexual coercion during the preceding year	Married women aged more than 20 years	Former or current husband	Mean ± SD: 35.85 ± 8.97	Junior and lower: 28.8% Senior: 22.0% College or higher: 48.9%
18 2021 [39]	Chengdu	Rural and urban	340	Self-administered questionnaire	Current Population Survey 2017	Experiences of violent behaviors (physical assault, psychological aggression, and sexual coercion) and gendered control	Married women	Former or current husband	Mean ± SD: 39.21 ± 8.43	Mean ± SD: 4.34 ± 1.67
19 2021 [40]	Jiangsu, Zhejian, Henan, Guizhou, Gansu, Sichuan	Rural and urban	2987	Self-administered questionnaire	Current Population Survey 2018	Experiences of violent behaviors (physical assault, psychological aggression, and sexual coercion), neglect and gendered control	Women who have/had an intimate partner	Former or current intimate partner	Mean ± SD: 36.5 ± 9.5	Primary: 25.1% Secondary: 41.9% Higher: 33.0%

**Table 3:** Variables associated with intimate partner violence (IPV) victimization for women in mainland China.

Variables	Details	Number of Studies Using This Variable	Number of Studies in Which the Variable as a Risk Factor Was Significant	References for Significant Studies
Individual risk factors				
Age				
	Older age	16	2	15, 16
	Younger age	16	2	2, 11
Woman’s age at marriage	22 years or lower	1	1	10
Couple’s age gap	Wide	1	1	5
Woman’s education level				
	Low	14	9	4, 5, 6, 8, 11, 12, 13, 15, 16
	High	14	2	2, 10
	Higher than husband	1	1	4
Partner’s education level	Low	7	3	3, 5, 11
Woman’s income	High	5	1	15
Partner’s income	Low	2	2	5, 1
Economic contribution to the family				
	Wife contributes more	2	2	6, 3
	Husband contributes more	3	2	6, 19
Couple’s original family’s economic status				
	Husband’s better	2	2	3, 5
	Wife’s better	1	1	3
Woman’s level of financial autonomy	Low	1	1	10
Woman’s employment status				
	Employed	5	3	6, 12, 14
	Industrial worker	1	1	16
	Job change in the past year	1	1	10
Partner’s employment	Unemployed/low occupational status	2	2	18, 19
Woman’s marital status				
	Cohabitation	2	1	16
	Remarriage	4	2	15, 17
	Divorced or in the divorce process	3	2	12, 14
Woman’s health status	Low	4	4	7, 8, 4, 6
Woman’s religious belief	Buddhism	1	1	4
Region				
	Rural areas	7	3	3, 6, 17
	Western area of China	3	3	3, 4, 19
	Middle area of China	2	1	3
Woman’s land rights status	No claim to contract land or residential land	2	2	5, 11
Woman’s status floating population	Yes/no local hukou (household registration)	4	3	6, 12, 14
Woman’s gambling	Sometimes or often	1	1	14
Husband’s gambling	Sometimes or often	4	3	7, 8, 14
Husband’s alcohol use	Yes	5	2	1, 14
Husband’s drug use	Yes	1	1	14
Woman’s media use	Less time spent reading paper books, more time spent online	1	1	12
Woman’s online activities	Watch movies or TV shows	1	1	12
Woman’s level of self-efficacy				
	Low	2	1	4
	High	2	1	7
Woman’s psychological well-being	Higher self-esteem	2	1	12
	Stronger feeling of loneliness or helplessness	2	2	12, 14
Relationship/family risk factors				
Power	Wife dominance	3	2	4, 6
Frequency of quarrels with husband	Sometimes or often	1	1	10
Level of woman’s marriage satisfaction	low	4	2	15, 17
Level of woman’s satisfaction with family living conditions	High	2	1	7
Woman’s experience with family-of-origin violence	Ever	3	2	7, 9
Spouse’s experience of family-of-origin violence	Ever	1	1	10
Years since woman’s marriage	Longer	8	3	6, 5, 11
Condition of children				
	Higher number	5	2	5, 8
	No children younger than six years	1	1	4
Family size				
	Bigger	2	1	3
	Smaller	2	1	8
Woman’s position in a family	low	2	2	4, 1
Woman’s social support				
	Big lending fund support network	1	1	8
	Woman involved with more types of nongovernmental organizations	1	1	4
	Small important decision-support network	1	1	8
	Low level	1	1	13
Family support				
	Low level of spouse’s support	1	1	8
	Low level of relative’s support	2	2	8, 10
	High level of children’s support	1	1	8
	High level of parents’ support	1	1	8
Society/Cultural/Attitudinal risk factors				
Woman’s satisfaction with social security	Low level of rural pension insurance	1	1	7
Woman’s willingness to move to an urban region	High	1	1	8
Regional sex ratio	Increased (more men)	1	1	5
Husband dominance	Yes	5	4	3, 4, 6, 19
Identification with traditional family culture/gender role	Yes	3	3	3, 8, 14
IPV justification	Yes	2	2	10, 14
Level of understanding of Women’s Rights Protection Law	Lower	1	1	4
